# Relating Pupil Dilation and Metacognitive Confidence during Auditory Decision-Making

**DOI:** 10.1371/journal.pone.0126588

**Published:** 2015-05-07

**Authors:** Karolina M. Lempert, Yu Lin Chen, Stephen M. Fleming

**Affiliations:** 1 Department of Psychology, New York University, New York, New York, United States of America; 2 Center for Neural Science, New York University, New York, New York, United States of America; 3 Department of Experimental Psychology, University of Oxford, Oxford, United Kingdom; Inserm, FRANCE

## Abstract

The sources of evidence contributing to metacognitive assessments of confidence in decision-making remain unclear. Previous research has shown that pupil dilation is related to the signaling of uncertainty in a variety of decision tasks. Here we ask whether pupil dilation is also related to metacognitive estimates of confidence. Specifically, we measure the relationship between pupil dilation and confidence during an auditory decision task using a general linear model approach to take into account delays in the pupillary response. We found that pupil dilation responses track the inverse of confidence before but not after a decision is made, even when controlling for stimulus difficulty. In support of an additional post-decisional contribution to the accuracy of confidence judgments, we found that participants with better metacognitive ability – that is, more accurate appraisal of their own decisions – showed a tighter relationship between post-decisional pupil dilation and confidence. Together our findings show that a physiological index of uncertainty, pupil dilation, predicts both confidence and metacognitive accuracy for auditory decisions.

## Introduction

The majority of decisions we make are accompanied by a sense of confidence. Whether we are making choices based on value (e.g., choosing a new TV set) or on perception (e.g., hailing an oncoming taxi on a foggy day), we possess metacognitive awareness of how good our decisions are, even in the absence of explicit feedback. Impaired awareness of uncertainty may underpin characteristics of neuropsychiatric conditions such as anxiety and obsessive-compulsive disorder [1, 2]. For example, decreased confidence in one’s own memory can lead to pathological “checking” behavior [3]. The ability to accurately appraise one’s uncertainty, known as “metacognitive” accuracy, is thus crucial for guiding adaptive behavior, particularly when direct feedback from the environment is unavailable.

People vary in their ability to construct these metacognitive estimates [4]. Despite the ubiquity of confidence in decision-making, how metacognitive evaluations are constructed is poorly understood. According to one view, uncertainty is an inherent property of the neural code underpinning perception, decision and action [5, 6] and may be tracked by neuromodulatory systems such as the locus coeruleus-norepinehrine (LC-NE) system [7]. However, the encoding of uncertainty is not itself metacognitive; instead, metacognition requires access to uncertainty signals to guide adaptive behavior such as explicit confidence reports [8, 9] or contingent decisions [10, 11]. Pupil dilation has previously been established as a physiological marker of decision uncertainty [12, 13], and is continuously elevated during the decision process [14]. Here we ask whether pupil dilation is also related to metacognitive estimates of confidence.

Participants carried out an auditory decision task in which objective difficulty was controlled, in order to isolate a relationship between metacognitive confidence and the pupil dilation signal. We additionally harnessed pupil dilation as a latent marker of the timing of confidence-related information in the brain. Specifically, it is unknown whether metacognitive estimates are based on information available at the time of decision (decisional locus), and/or on information available after the decision has been made (post-decisional locus). In decisional locus models, confidence ratings are based on the same evidence that is used to make the judgment [11, 15–18], with choices and confidence ratings tapping the same latent process. While this theory is intuitively attractive, there is evidence that a post-decisional component may also contribute to confidence ratings. For instance, we occasionally “change our mind” even after a motor program has been initiated [19], and confidence judgments change significantly when we are given less time to make them ([20]; see [21] for review). In post-decisional locus models (e.g., two-stage dynamic signal detection theory; [22]), confidence ratings depend on continued processing of evidence post-decision; that is, while cues used for making a perceptual decision may be incorporated into the confidence judgment, there may also be additional processing that contributes to confidence after the initial judgment has been made.

Although behavioral evidence for post-decisional locus models exists, there is a lack of physiological or neural evidence demonstrating a post-decision contribution to confidence in humans. We hypothesized that the presence of a correlation between pupil dilation and confidence at different time points in the decision process would shed light on the processing locus for judgments of confidence. Furthermore, this locus may vary depending on the accuracy of people’s metacognitive judgments. For example, subjects with more accurate metacognition may continue to reflect on uncertainty-related signals after a decision is made; alternatively, post-decision noise may corrupt confidence estimates and lead to poorer metacognitive assessments [8, 21].

We examined the relationship between pupil dilation signals at different time points during the decision process and the generation and accuracy of metacognitive confidence judgments in an auditory decision task. A general linear model (GLM) analysis allowed the disentangling of different temporal components of the neural input to the sluggish system controlling pupil dilation, in an analogous manner to an analysis of the blood oxygen level-dependent (BOLD) signal in functional magnetic resonance imaging (fMRI) data [14]. By presenting auditory stimuli, we ensured that pupil responses were unconfounded by changes in visual stimulation. Furthermore, by adjusting task difficulty online we were also able to quantify each individual’s metacognitive judgments unconfounded by differences in task performance [23].

## Materials and Methods

### Participants

Forty-two individuals completed an auditory perceptual decision task while their pupil dilation response was monitored. Thirty-five subjects were included in final analyses (23 F, 12 M; mean age = 22.94; SD = 3.46; n = 2 were excluded for insufficient stabilization of psychophysical performance; n = 5 were excluded for unreliable eye tracking). Past studies of pupil dilation have drawn conclusions from approximately 20–30 participants (e.g., [12, 24]). We used a staircase procedure to equate performance among subjects (see Analyses below) resulting in approximately the same performance level for all participants (between 69 and 75% correct).

### Ethics Statement

Approval was obtained from The University Committee on Activities Involving Human Subjects at New York University (HS#13–9582), and all participants signed a printed consent form before the experiment.

### Task

**Fig 1:**
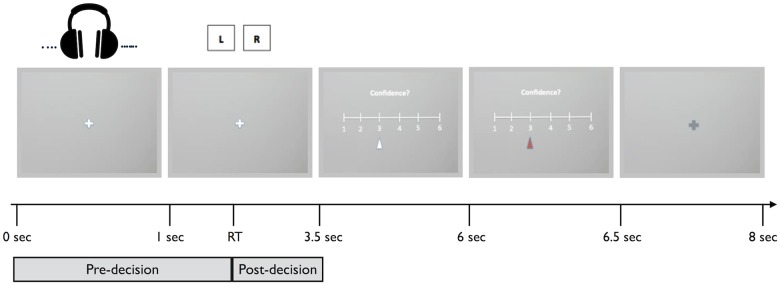
Auditory decision task layout.On each trial, participants heard a series of clicks presented binaurally for 1 second while pupil diameter was measured. Participants made a button press to indicate in which ear they had heard more clicks, followed by a confidence rating, before proceeding to the next trial.

The task was a two-alternative forced choice auditory version [25] of the random-dot motion task [26], presented using the Matlab Psychophysics Toolbox (MathWorks, Natick, MA). At the beginning of the experiment, participants put on headphones and rested their chin on a chin rest so that pupillometric data could be collected using EyeLink 1000 equipment (SR Research, Ontario, Canada). Subjects were seated 55 cm from the computer screen, and pupil diameter was sampled at 250 Hz. At the onset of each trial, participants heard a series of clicks played binaurally through headphones for 1 second. They were then required to decide whether they heard more clicks in the left or right ear. They indicated their judgment by pressing the left or right arrow key on a standard QWERTY keyboard. Participants were asked to keep their eyes on a fixation cross in the center of the screen during stimulus presentation and decision. 3.5 seconds after stimulus onset the confidence scale appeared (if response times were longer than 3.5 seconds, the confidence scale appeared immediately after the response; these trials comprised ~5% of all trials). The scale ranged from 1 on the left (low relative confidence) to 6 on the right (high relative confidence). Participants were asked to use the left and right arrow keys to move the cursor along the scale until they reached a value on the scale that corresponded to how confident they felt about their judgment on that trial. After 3 seconds had elapsed, the participant’s response was recorded. The cursor remained on the recorded confidence response for 0.5 seconds; then the confidence scale disappeared from the screen. The initial placement of the cursor along the scale was random so that responses were not biased toward any part of the scale. In addition, participants were encouraged to treat the confidence scale as a relative scale, and to use the whole scale throughout the experiment. After a 1.5-second inter-trial interval, the next trial began (see Fig 1 for task layout).

The auditory stimuli consisted of Poisson-distributed click trains. Each click was a 23 ms burst of white noise sampled at a rate of 44 kHz. The overall click rate was set to 200 Hz. On each trial, the number of clicks played to each ear varied depending on the ratio of click rates for the correct and incorrect response. The ratio was adjusted on each trial using a 1-up 2-down staircase procedure [27], which at the limit converges on 71% accuracy. Our aim in using this staircase procedure was to equate objective perceptual performance across individuals, leaving quantification of metacognitive ability unconfounded by differences in performance. We used the ratio of clicks for the correct response to clicks for the incorrect response as our measure of difficulty for each trial. When this ratio is greater, the trial is easier. There were 180 trials total, divided into 3 blocks. Participants were allowed to take a break between blocks, and the eye tracker was re-calibrated in between each block.

### Analyses

#### Pupil dilation preprocessing

Pupil diameter data were analyzed using Matlab 7.11 (MathWorks, Natick, MA). Eye-blinks were categorized as pupil dilation changes that transpired too quickly to represent actual pupil dilation; they were removed using linear interpolation. We baseline-corrected the pupil data by dividing each pupil diameter measurement by the average pupil diameter 1 second prior to each trial, as done previously [14, 28, 29]. All analyses were performed on the preprocessed pupil diameter data.

#### GLM analysis

In order to best determine the timing of confidence-related activity in the pupil data, we constructed a general linear model (GLM), with the following predictors (as individual 1s in a series of 0s): 1) stimulus onset, 2) a sustained component (boxcar) during the decision period (from the onset of the stimulus to the time of response), 3) response onset, and 4) a sustained component during the post-decision period (from the response time to the onset of the confidence rating screen). By constructing regressors in this way, we allow for temporally extended pupil responses when decision time is greater. We omitted the post-decision period regressor on the small fraction (~5%) of trials on which participants took longer than 3.5 seconds from stimulus onset to respond, since on these trials, participants saw the confidence screen immediately after they made their response. We normalized the boxcar regressors by dividing the height of the boxcar by its length such that these regressors had the same norm as the stick regressors. In addition, we included each of these regressors in the model parametrically modulated by 1) difficulty (i.e., the ratio of correct to incorrect clicks on each trial) and 2) the confidence rating on each trial. We z-scored all confidence ratings to account for individual differences in use of the confidence scale. Therefore, our final model contained twelve predictors: the four temporal components, the four temporal components modulated by difficulty, and the four temporal components modulated by confidence. Each regressor was then convolved with a canonical pupil response function (described in [14]):

$$h(t)=s*t^{w}e^{\left(-t*w/t_{\max}\right)}$$

where w is the width and t_max_ is the time-to-peak (in milliseconds) of the impulse response function. We used canonical values of these two parameters (see [14, 30]): w = 10.1; t_max_ = 930 ms. We scaled the function by a factor *s* = 1/10^27^ (as in [31]), and resampled the predicted response to match the sample rate of the data (250 Hz). The convolved regressors were horizontally concatenated into a design matrix. Multiple regression yielded the best-fitting beta weights for each regressor, separately for each subject. Statistical inference at the population level was carried out by comparing these values to the null hypothesis that average beta weights are zero using one-sample t-tests.

#### Regression model (performance)

To check for stabilization of performance, data were divided into five blocks. The first block of trials was excluded for each participant to permit staircase stabilization. A one-way ANOVA on click rate ratio revealed no significant differences in stimulus difficulty over the remaining four blocks (*F*_*(3*,*136)*_ = 0.114; *p* = 0.95).

To quantify the link between confidence and task performance we set up a multiple logistic regression model predicting accuracy (1 = correct; 0 = incorrect) from z-scored confidence ratings and difficulty (click rate ratio), with standard errors clustered by subject.

#### Metacognitive accuracy

To quantify metacognitive accuracy for each individual, we constructed type II receiver operating characteristic (ROC) curves as described previously (see [23, 32] for further details). The area between the major diagonal and an individual’s type II ROC curve is a measure of the ability to link confidence to performance on the perceptual decision-making task (A_roc_). To construct the ROC we binned each individual’s confidence ratings into five quintiles. *h*_*i*_ = p(confidence = *i*|correct) and *f*_*i*_ = *p*(confidence = *i*|incorrect) were calculated for all *i*, transformed into cumulative probabilities and plotted against each other. ROC curves were anchored at [0,0]. The intuition behind this analysis comes from signal detection theory (SDT). Conventional applications of SDT assess detection performance by comparing the proportion of “hits” and “false alarms” in a stimulus detection task. By applying the logic of SDT to metacognition (“Type II” SDT), a “hit” becomes a high confidence response after a correct decision, and a “false alarm” is a high confidence response after an incorrect decision. An ROC curve that bows sharply upwards indicates that the probability of being correct rises rapidly with confidence; conversely, a flat ROC function indicates a weak link between confidence and accuracy. We note that type II ROC area is affected by type 1 *d’* and criterion [33, 34]. Here, use of a 2-alternative forced choice (2AFC) design and a continuous staircase permitted tight control over these factors, dissociating metacognitive accuracy from task performance.

#### Control for eye movements

It has recently been demonstrated that gaze direction and eye movements may produce systematic errors in pupil diameter measurements [35]. To control for potential eye movement artifacts, we instructed participants to fixate on the center of the screen throughout the decision period. In addition, we re-calibrated the eye tracker at the beginning of each block and used a gaze-contingent display at the start of each block to ensure that the fixation point did not shift between blocks. Nevertheless, participants did make some minor eye movements. To control for these fluctuations, we calculated a “deviation index” for each trial for each subject. First, we calculated the Euclidean distance of the eye from the fixation point for each recorded pupil diameter sample. Then, we normalized these distances by dividing each by the median distance (the median was used as the measure of central tendency due to the skewed nature of these distributions). Finally, we averaged these values in order to determine a “deviation index” for each trial. We constructed an additional general linear model, in which each of the temporal regressors was parametrically modulated by the deviation index on that trial (thereby adding an additional four regressors to our main model).

## Results

**Fig 2:**
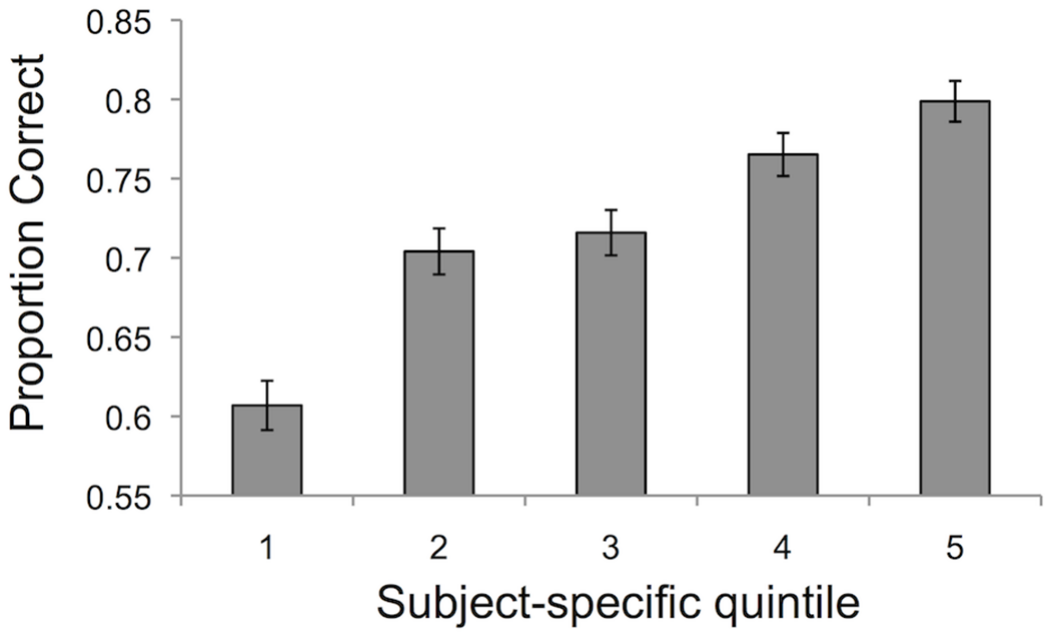
Proportion correct as a function of subject-specific confidence quintile. Confidence ratings increase with performance in the task, demonstrating above-chance metacognitive accuracy (5 = highest fifth of confidence ratings; 1 = lowest fifth of confidence ratings).

Participants carried out a near-threshold auditory discrimination task while pupil dilation responses were measured. Following each auditory decision, participants were asked to provide explicit judgments of confidence. Average performance ranged from 69–75% correct, and Fig 2 shows that post-decision confidence ratings increased with task performance. Accordingly, in a logistic regression, confidence positively predicted accuracy (1 = correct; 0 = incorrect) even when controlling for stimulus difficulty (*β* = 0.432; Wald *X* ^*2*^ = 92.28; *p* = 0.0009). Thus on average participants have good metacognitive accuracy, monitoring fluctuations in task performance in the absence of feedback. Median average response time from stimulus onset was 1860 ms (range: 1405–3185 ms).

**Fig 3:**
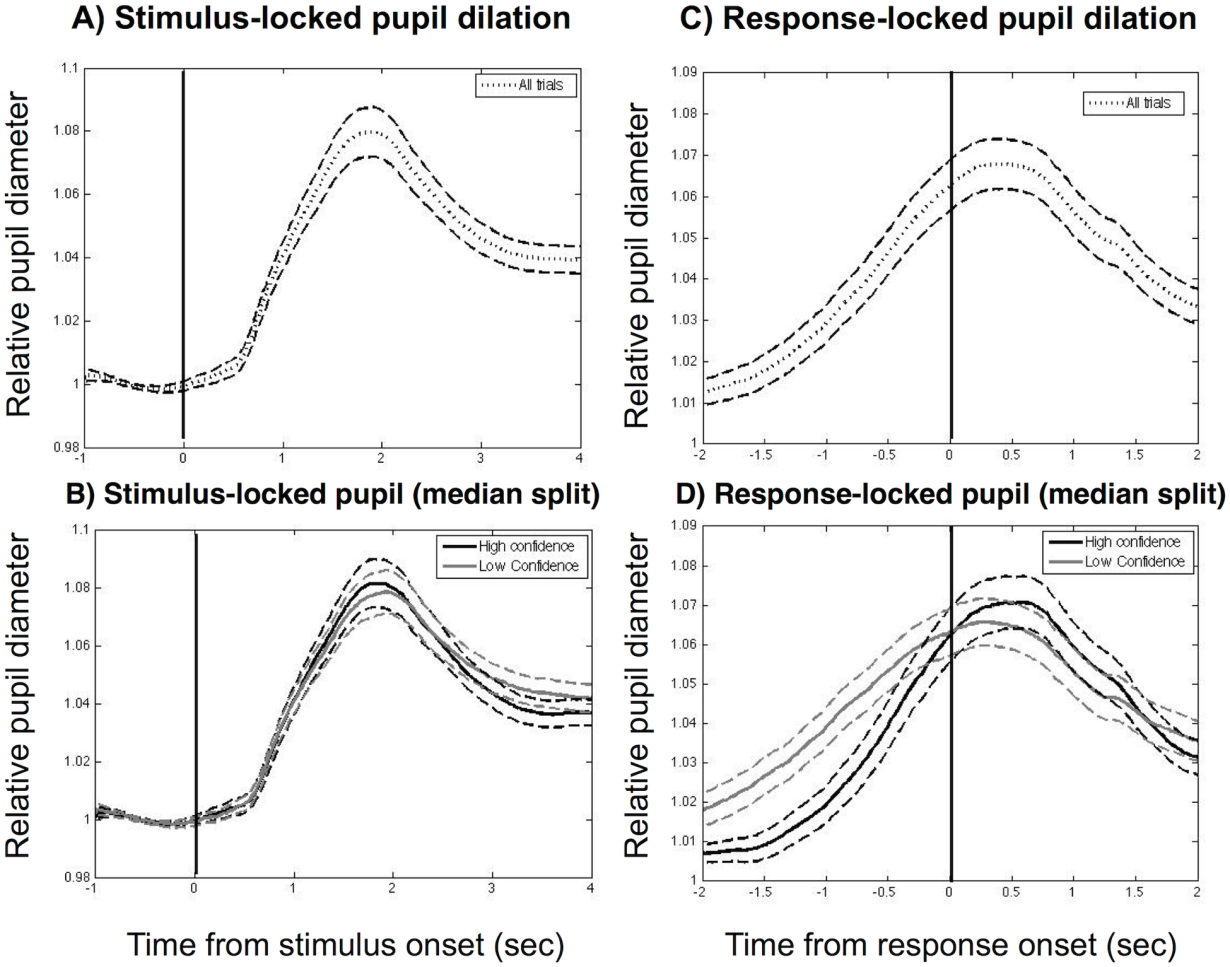
Stimulus-locked and response-locked pupil dilation response. (A, B) Time course of pupil diameter averaged over all subjects (n = 35), aligned to stimulus onset for (A) all trials and (B) low and high confidence trials (median split). (C, D) Time course of pupil diameter aligned to response onset for (C) all trials and (D) low and high confidence trials (median split). Dashed lines represent standard error of the mean. Black vertical lines indicate timings of stimulus onset (A and B) and response onset (C and D).

To visualize the time course of the pupil response, we binned the pupil response into 20 ms bins and plotted both the stimulus-locked and response-locked responses (Fig 3a and 3c). These traces show, first, that there was an increase in pupil diameter at the time of stimulus onset, which remained elevated throughout the decision period. Second, there was an increase in pupil dilation around the time of the motor response. Separating the pupil response into low and high confidence trials, we observed a greater increase in pupil dilation for low confidence trials that persisted until the response (Fig 3b and 3d).

To further quantify these effects, we modeled the time course as a linear superposition of four temporal components: two transients (one at stimulus onset and one at response onset), and two boxcar regressors (one pre-decision and one post-decision), convolved by a canonical pupil response function (see Materials and Methods). Regressors for each of these components modulated by difficulty and confidence rating were also included.

**Fig 4:**
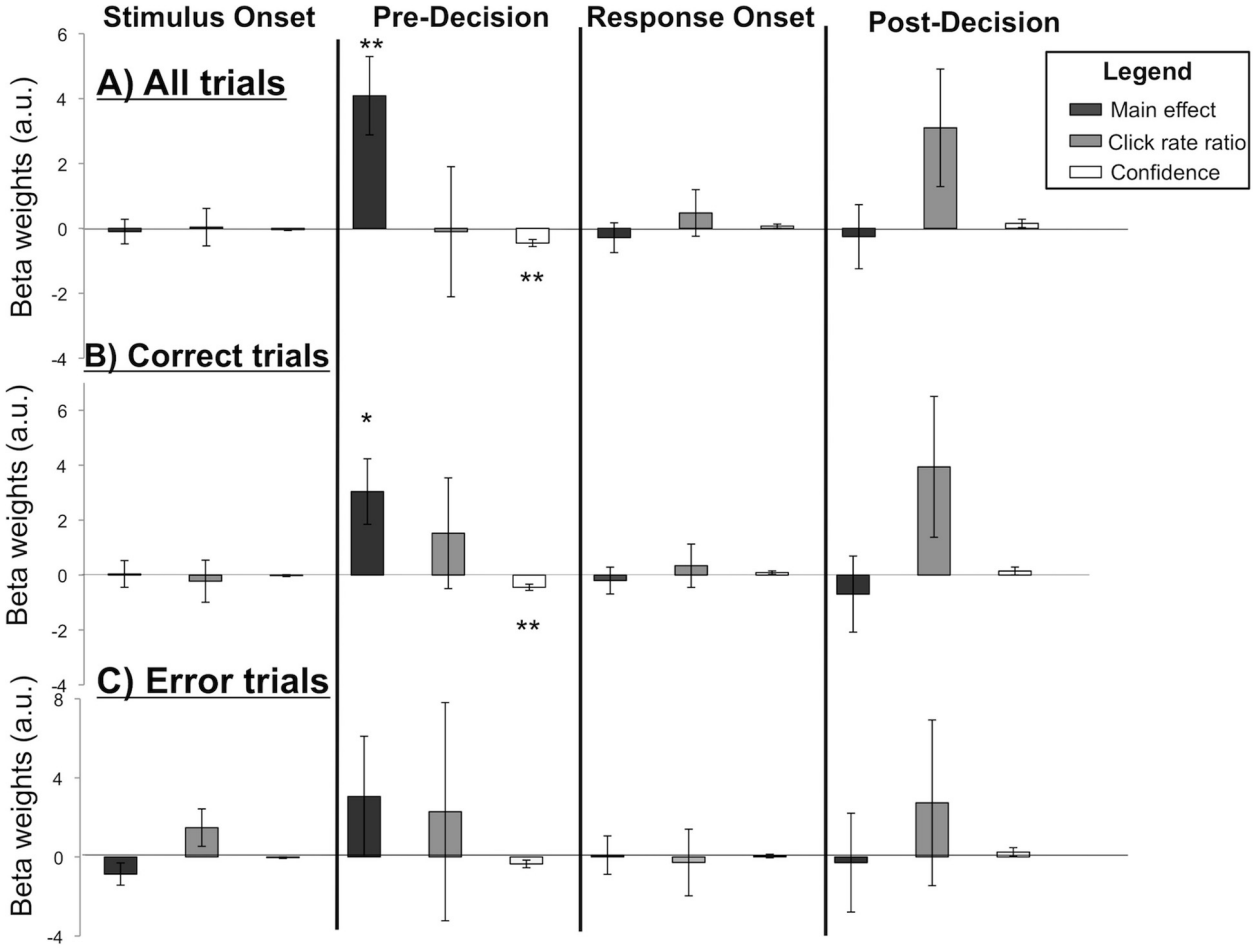
Results of a general linear model analysis of the pupil dilation response. Each panel shows the average of best-fitting beta weights for each of four temporal components (stimulus onset, pre-decision, response onset, and post-decision), as well as the four components parametrically modulated by difficulty (click rate ratio; when this value is higher, the choice is easier) and confidence, across all subjects (n = 35) for all trials (A), correct trials (B) and error trials (C). Error bars represent SEM; * = significant at *p* < 0.05 (two-tailed t-test); ** = significant at *p* < 0.01.

Average beta weights for each of our twelve predictors are plotted in Fig 4a. First, we found a significant positive main effect of the pre-decision period boxcar regressor (*t*_*34*_ = 3.39; *p* = 0.002), but no significant effect of the stimulus onset regressor (*t*_*34*_ = -0.26; *p* = 0.80), the response onset regressor (*t*_*34*_ = -0.62; *p* = 0.54) or the post-decision boxcar regressor (*t*_*34*_ = -0.26; *p* = 0.80). This pattern replicates previous findings of a sustained increase in pupil dilation during decision-making [14]. Crucially, we found that confidence negatively predicted the pupil dilation response in the pre-decision period (*t*_*34*_ = -4.18; *p* < 0.001). In other words, lower confidence is associated with greater pupil dilation during the decision period, after controlling for objective stimulus difficulty. This result also held when we controlled for eye movements during the trial (*t*_*34*_ = -3.95; *p* < 0.001). Confidence did not predict pupil dilation at any other point during the time course of the trial (at stimulus onset: *t*_*34*_ = -1.37, *p* = 0.18; at response onset: *t*_*34*_ = 1.13, *p* = 0.27; in the post-decision period: *t*_*34*_ = 1.23, *p* = 0.23).

We considered that the negative relationship between confidence and pupil dilation pre-decision may be driven by signals on error trials, as pupil dilation responses have previously been associated with errors in a variety of tasks (e.g., [36]). We therefore carried out a regression analysis separately for correct and error trials (Fig 4b and 4c). Consistent with the previous analysis, on correct trials (~70% of all trials) we found a significant positive main effect of the pre-decision boxcar regressor on pupil dilation (*t*_*34*_ = 2.55; *p* = 0.015) as well as a significant negative effect of confidence (*t*_*34*_ = -3.96; *p* < 0.001). On error trials, which comprised a smaller subset of all trials, we observed a similar pattern of results which did not reach statistical significance, possibly due to lack of power (pre-decision boxcar main effect: *t*_*34*_ = 1.00; *p* = 0.32; pre-decision boxcar modulated by confidence: *t*_*34*_ = -1.85; *p* = 0.07).

We next turned to the functional consequences of the link between pupil dilation and confidence by quantifying each subject’s metacognitive accuracy. Metacognitive accuracy reflects the link between objective performance—the trial-by-trial likelihood of giving a correct response—and confidence. There was considerable variation across individuals in metacognitive accuracy (A_roc_ = 0.48 to 0.76), despite underlying task performance being held approximately constant (percent correct: 69 to 75%); furthermore, these measures were uncorrelated (*r* = 0.32, *p* = 0.07). To establish whether the relationship between pupil dilation and confidence was associated with metacognitive accuracy, we conducted a Pearson correlation between each of the four confidence-related beta weights and the metacognitive accuracy score (A_roc_) for each subject, separately for correct and error trials. Bonferroni correction was applied to control for multiple comparisons. Decomposing the data in this way ensured that any non-specific pupil dilation response to errors would not induce a spurious correlation with metacognitive accuracy.

**Fig 5:**
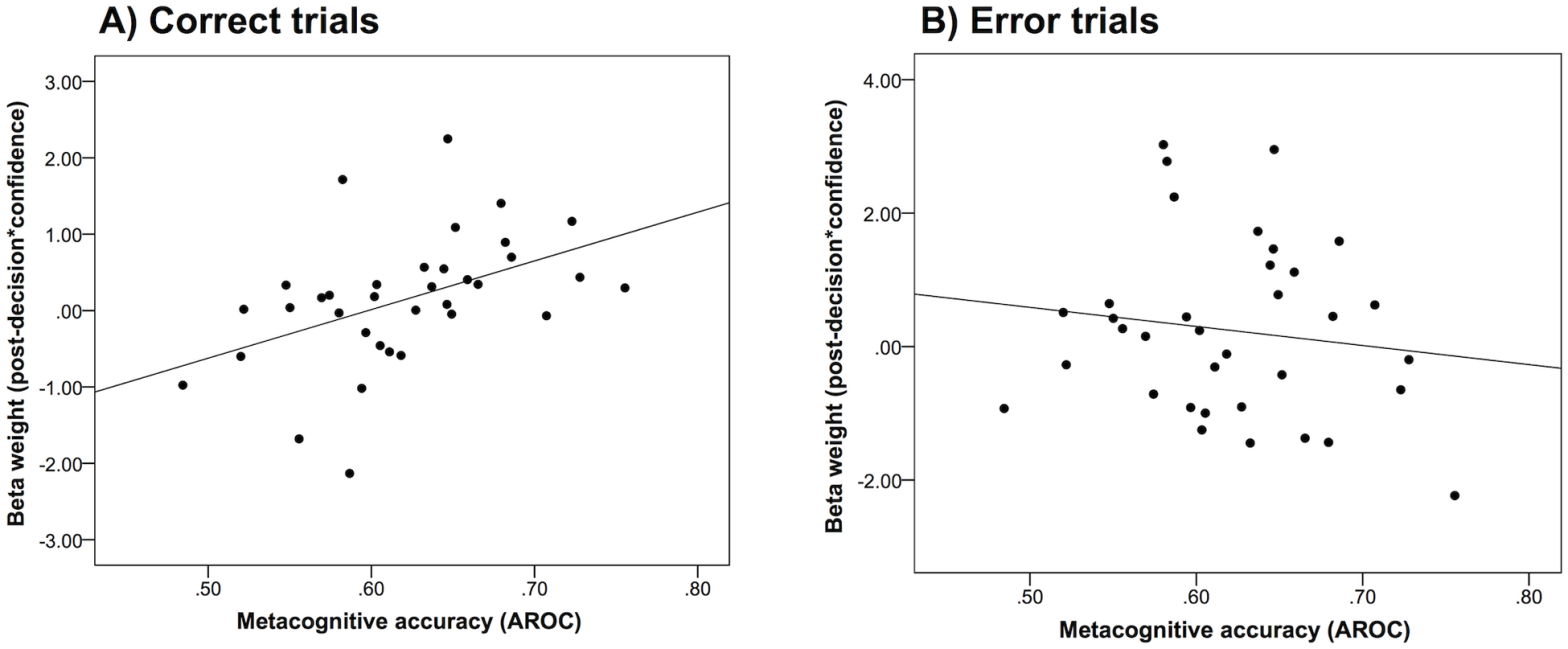
Relationship between metacognitive accuracy, as measured by area under the Type-II ROC curve (A_roc_), and the effect of confidence on post-decision pupil dilation for correct trials (A) and for error trials (B). Individuals with better metacognitive accuracy show greater confidence-related pupil dilation during the post-decision period on correct trials (n = 35; *r* = 0.46; *p* = 0.005). This relationship was not found on error trials (*r* = -0.14; *p* = 0.44).

On correct trials, we found that metacognitive accuracy moderated the relationship between confidence and pupil dilation responses during the post-decision period (correlation with post-decision boxcar beta: *r* = 0.46; *p* = 0.005; Fig 5a). This effect remained when controlling for eye movements (*r* = 0.46; *p* = 0.005). No relationship was found between metacognitive accuracy and confidence-related pupil dilation pre-decision (correlation with pre-decision boxcar beta: *r* = 0.13, *p* = 0.45; controlling for eye movements: *r* = 0.17, *p* = 0.34). However, caution is warranted when interpreting the difference between pre- and post-decision effects on metacognitive accuracy as the difference between these correlation coefficients was not significant (Fisher’s r-to-z transformation: *z* = -1.45; *p* = 0.15). The correlation between A_roc_ and confidence-related pupil dilation was not significant for either of the onset regressors (stimulus onset: *r* = 0.08; *p* = 0.63; response onset: *r* = -0.12; *p* = 0.47). Finally, no relationship was found at any time point for incorrect trials (stimulus onset: *r* = -0.14; *p* = 0.43; pre-decision boxcar: *r* = 0.008; *p* = 0.96; response onset: *r* = 0.03; *p* = 0.85; post-decision boxcar: *r* = -0.14; *p* = 0.44; Fig 5b).

These results suggest that metacognitive accuracy is specifically enhanced in subjects who show a tight pupil-confidence relationship after a decision is made, and support an additional post-decisional contribution to confidence judgments and metacognition.

## Discussion

How individuals construct metacognitive estimates of confidence is poorly understood. Metacognition requires access to uncertainty signals to guide adaptive behavior such as explicit confidence reports or contingent decisions. Pupil dilation has previously been established as a physiological marker of decision uncertainty [12, 13], and is continuously elevated during the decision process [14]. Pupil dilation is affected by both noradrenergic (NE) and cholinergic (ACh) neuromodulation [37, 38, 40], and activity in the locus coeruleus (LC), the major noradrenergic nucleus in the brain, also reflects uncertainty during learning [39]. The current study demonstrates that non-luminance mediated changes in pupil dilation during a pre-decision period correlate with metacognitive confidence, even when objective stimulus difficulty is controlled. Specifically, a greater pupil dilation response was associated with lower confidence, consistent with findings linking pupil dilation and uncertainty [12, 13]. This relationship was also observed when correct trials were analyzed separately, ensuring that effects of confidence were not confounded by error-related pupil dilation responses [36].

We additionally harnessed pupil dilation as a latent marker of the timing of metacognitive processes. In particular, it is unclear whether confidence judgments are computed in parallel to the decision (decisional locus) or if confidence judgments are based on continued processing after the decision (post-decisional locus). Our results shed light on this issue. Consistent with a decisional locus account, pre-decision pupil dilation negatively predicted confidence. At the group level, there was no significant relationship between confidence ratings and post-decision pupil dilation. However, subjects with better metacognitive accuracy showed a stronger link between post-decision pupil dilation and confidence. This finding suggests that continued processing after a choice is made also contributes to the accuracy of metacognitive judgments. This is consistent with theories proposing post-decision accumulation of evidence for confidence [8, 22] and suggests that metacognitive accuracy may depend on a late-stage integration of uncertainty-related neural responses. The stronger link between pupil dilation and confidence in high-metacognition subjects is also consistent with interoception being an important determinant of metacognitive ability [41]. Together our findings support a contribution of both pre- and post-decision factors to the construction of metacognitive confidence.

Some limitations of the current study warrant mention. First, we did not manipulate decision time—participants could choose to respond whenever they wished. It is possible that some participants constructed a confidence judgment before they made a perceptual decision under these circumstances, thereby reducing their reliance on post-decisional information. Future studies should manipulate or restrict decision time to determine if this influences the timing of pupil dilation and its link to metacognitive processing. Second, by design, error trials were less numerous, and therefore our power to detect confidence-related effects on error trials was lower than on correct trials. It would be of interest to determine whether qualitatively distinct characteristics of the pupil dilation response on error trials contribute to metacognition in future work.

Uncertainty is a pervasive feature of our environment—neural processing is noisy and external events are unpredictable. Therefore, the ability to accurately appraise one’s uncertainty is integral to making good decisions. When uncertainty is high, for example, we should boost our learning rates and direct our attention to gathering more information [42–44]. The sources of evidence we use to make these judgments remain poorly understood. Here we show that a physiological index of uncertainty (pupil dilation) predicts metacognitive confidence in auditory decision-making. These findings have implications for models of decision-making and metacognition, as well as for disorders such as anxiety or obsessive-compulsive disorder in which awareness of uncertainty may be compromised.
